# New Insights into Phloem Unloading and Expression of Sucrose Transporters in Vegetative Sinks of the Parasitic Plant *Phelipanche ramosa L*. (Pomel)

**DOI:** 10.3389/fpls.2016.02048

**Published:** 2017-01-09

**Authors:** Thomas Péron, Adrien Candat, Grégory Montiel, Christophe Veronesi, David Macherel, Philippe Delavault, Philippe Simier

**Affiliations:** ^1^Laboratoire de Biologie et de Pathologie Végétales EA 1157, SFR 4207 QUASAV, Université de NantesNantes, France; ^2^UMR 1345 IRHS, SFR 4207 QUASAV, INRA, Agrocampus-Ouest, Université d'AngersBeaucouzé, France

**Keywords:** broomrape, parasitic weeds, phloem unloading, sink strength, sink-source relationships, sucrose carriers

## Abstract

The plant-parasitic plant interaction is a interesting model to study sink-source relationship and phloem unloading. The parasitic plants, such as the achlorophyllous plant *Phelipanche ramosa*, connect to the host phloem through the haustorium and act as supernumerary sinks for the host-derived photoassimilates, primarily sucrose. The application of the fluorescent symplastic tracer, carboxyfluorescein (CF) derived from carboxyfluorescein diacetate (CFDA), to the leaves of the host plant (*Brassica napus*) showed direct phloem connections at the host-parasite interface. These experiments also evidenced the dominant apoplastic pathway for phloem unloading in major vegetative sinks of the parasite, including tubercles and shoots, except the adventitious root apices. The CF experiments showed also the symplastic isolation of the phloem tissues from the sink tissues in tubercle and shoot of the parasite, then suggesting the pivotal role of sucrose transporters in sucrose unloading in *P*. *ramosa* sinks. Three cDNAs encoding sucrose transporters (*PrSUT*) were isolated from the parasitic plant. *PrSUT1* transcripts accumulated at the same level in the tubercle throughout the parasite growth while a significant increase in transcript accumulation occurred after emergence in the flowering shoot, notably in the growing apical part. The *in situ* hybridization experiments revealed the *PrSUT1* transcript accumulation in the mature phloem cells of both subterranean and flowering shoots, as well as in shoot terminal sinks corresponding to apical meristem, scale leaf primordia and immature vasculature. The transient expression experiments in *Arabidopsis* protoplasts showed that PrSUT1 was localized at the plasma membrane, suggesting its role in phloem functioning and sucrose uptake by the sink cells in *P*. *ramosa*. Conversely, the *PrSUT2* transcript accumulation was constantly low in tubercles and shoots but *PrSUT3* transcripts accumulated markedly in the subterranean and flowering shoots, in concordance with the *PrSUT3* mRNA accumulation in multiple sink areas including apical meristem, scale-leaf primordia, immature vasculature and even storage parenchyma. However, the *PrSUT3* transcripts did not accumulate in the mature phloem cells. The transient expression experiments in *Arabidopsis* protoplasts suggested a tonoplast localization of PrSUT3, for which nevertheless the involvement in intracellular sucrose transport needs clarification.

## Introduction

Sucrose is the main photosynthetic product in higher plants and its long-distance translocation through the phloem from source organs to sink organs enables cell growth and resource storage in sink organs. Sucrose phloem unloading is extensively studied, considering the important issues of understanding and controlling the development of sink organs of economic interest for agri-food industry, such as tubers, fruits and seeds (Zhang et al., 2006; Yadav et al., 2015). The symplastic pathway is characterized by sucrose release from phloem cells into sink cells through plasmodesmata and symplastic *continuum*. Sucrose unloading is controlled by the decreasing gradient of sucrose concentration between phloem and sink cells triggered by the intensive sucrose metabolism and/or sucrose accumulation into the vacuoles in sink cells. In the apoplastic pathway, sucrose is transported from the phloem cells into the apoplast and is either uptaken by plasma membrane-localized sucrose transporters of the sink cells or hydrolyzed by apoplastic invertase into hexoses, which are imported into the sink cells by plasma membrane-localized hexose transporters. Besides, some SWEET proteins, corresponding to mono—and disaccharide facilitators, have been recently identified as the sucrose effluxers into the apoplast (Chen, 2014; Patil et al., 2015). Apoplastic unloading is particularly relevant in growing fruits to prevent symplastic backflow as the fruits accumulate large amounts of sugars (Jin et al., 2009).

Sucrose transporters (SUT proteins) are involved in sucrose phloem loading in the source organs, sucrose retrieval by the sieve elements during phloem translocation, and sucrose uptake and intracellular transport in the sink cells (Kühn and Grof, 2010; Lemoine et al., 2013). Among the *SUT* (or *SUC)* genes and proteins characterized in plants, most correspond to plasmalemma-specific or tonoplastic specific transporters utilizing the proton motive force for sucrose/H^+^ symport into the cytosol from the apoplast or the vacuole, respectively (Eom et al., 2011). Beside, some vacuolar sucrose transporters should act as sucrose/H+ antiporters for sucrose import into vacuoles in sucrose accumulating species, however the genes encoding these proteins remain to be identified (Getz and Klein, 1995; Kühn and Grof, 2010). For example, AtTMT is a proton-coupled antiporter capable of high-capacity loading of glucose and sucrose into the vacuole (Schulz et al., 2011).

The parasitic plants represent a particular group of higher plants due to either their ability (facultative parasites), or their obligation (obligate parasites) to parasitize another plant to fulfill their development and reproductive functions. Some of them exhibit a weedy behavior in grasslands and crops and are major pests which are difficult to control (Parker, 2009). Among these harmful parasitic weeds, the obligate root parasitic plant *Phelipanche ramosa* (syn. *Orobanche ramosa*) is widely distributed in the Mediterranean region and parasitizes many cultivated species including tobacco, tomato, hemp and oilseed rape. Following germination and seedling attachment to host roots through the haustorium, a specific organ which connects the host and the parasite phloem tissues (Yoshida et al., 2016), the parasitic plant acts as a strong competitive sink for water, growth regulators and nutrients especially photoassimilates (Hibberd and Jeschke, 2001). Then it develops a tubercle and in turn a subterranean shoot. Flowering and self pollinization occur rapidly after shoot emergence from the soil.

In this parasitic relationship, sucrose is thus the major organic compound transferred from the host to the parasite (Aber et al., 1983; Abbes et al., 2009). Consequently, sucrose transfer at the host-parasite interface, in addition to sucrose phloem unloading in the sink tissues of tubercle and shoot represent key processes in the parasite growth. Although the understanding of the mechanisms and regulation are still unknown, it has been shown that sucrose phloem unloading in *P. ramosa* leads to a strong sugar enrichment in the tubercle and primarily in the shoot. Both organs elaborate a complex carbohydrate pattern with very low sucrose levels in contrast to high hexose, mannitol and starch levels (Singh et al., 1968; Delavault et al., 2002). Interestingly, such a sugar pattern is common in broomrapes (*Orobanche* and *Phelipanche* species) and reveals the high activity in sucrose degradation of those parasites (Harloff and Wegmann, 1993; Abbes et al., 2009; Aly et al., 2009). The genes encoding sucrose-degrading proteins were characterized in *P. ramosa*. Among the five invertase genes and two sucrose synthases (*Sus*) genes identified, *PrSai1* was shown to encode a putative vacuolar invertase protein and to be mostly expressed in the tubercle and especially in the shoot during growth. Indeed, both organs display a dominant PrSAI1 activity. These findings suggest that PrSAI1-mediated sucrose hydrolysis in the vacuoles of the sink cells takes a central place in sucrose phloem unloading in the parasitic plant (Draie et al., 2011). *PrSus1* was shown to encode a sucrose synthase and its transcripts accumulate in the young xylem cells of tubercle, suggesting that PrSUS1 is involved in cellulose synthesis and xylem maturation (Péron et al., 2012).

The host plant-parasitic plant interaction in which the vegetative sinks of the parasitic plant represent the dominant sinks of the interaction is thus a particular and interesting model for studying sucrose phloem unloading in plants. The present study focuses on the oilseed rape–*P*. *ramosa* interaction and the use of a fluorescent dye as symplast tracer provides evidence that the host phloem and the parasite phloem tissues are symplastically connected within the haustorium. The confocal laser scanning microscopy (CLSM) analysis highlights that most of the vegetative sinks of the parasitic plant are symplastically isolated from the phloem network, showing that nutrient (especially sucrose) phloem unloading in those sink areas is apoplasmic. In this context, three sucrose transporter encoding cDNAs were isolated from *P. ramosa* (*PrSUTs*). The expression pattern analysis in addition to the *in situ* hybridization experiments and the transient expression assays in *Arabidopsis thaliana* protoplasts suggest the dominant role of the plasmalemma-specific transporter PrSUT1 in phloem functioning and sucrose import into sink cells of the shoot in *P. ramosa*. Previous reports underlined the preponderant role of the vacuolar invertase PrSAI1 in sucrose utilization in the sink cells of the parasite (Draie et al., 2011; Péron et al., 2012), then requiring the involvement of a tonoplastic sucrose transporter upstream for the sucrose influx into the vacuole. The present study suggests the tonoplast location of PrSUT3, nevertheless its involvement in the intracellular sucrose transport in sink cells still needs to be clarified.

## Materials and methods

### Plant materials

*Phelipanche ramosa* seeds were collected in an infested oilseed rape field (sampling site: Saint-Jean-d'Angely, France, in 2005) and stored in the dark at 25°C until use. Oilseed rape (*Brassica napus*, var. Campo)-*P*. *ramosa* L. Pomel interactions were grown in rhizotrons, as described by Gauthier et al. (2012). Various developmental stages of *P*. *ramosa* seedlings were harvested, including young tubercles developing numerous adventitious roots (stage III, phenotype “spider”), tubercles with a growing subterranean shoot (stage IV) and tubercles with emerged and flowering shoot (stage V) (Draie et al., 2011). The plant material was immediately frozen in liquid nitrogen and stored at −80°C prior to RNA extraction. The *in situ* hybridization experiments were performed on extemporaneously harvested tissues following paraformaldehyde fixation.

### Confocal laser scanning microscopy (CLSM)

Two hundreds of μl of 5(6) carboxyfluorescein diacetate (CFDA) (CFDA, Sigma-Aldrich, St. Louis, MO, USA) in an aqueous solution (1:20 dilution of a 6 mg/ml stock in acetone), was loaded for 2 h on an abraded mature leaf of the parasitized host plant. First, CF transfer in the parasitic plant (stages III–V) was monitored by fluorescence Leica MZ FL III binoculars (480/535 nm excitation/emission). Then parasite tubercles and shoots (stages III and IV) were subsequently cut using a vibratome (HM 650V, Microm) into transverse or longitudinal sections (200 μm thick) and soaked immediately into immersion oil to prevent dye loss. The tissue distribution of CF was monitored using a Nikon A1 confocal laser scanning microscope (x4 objective). The images were acquired using a 488 nm laser for excitation and the emission was collected via a photomultiplier through band-pass filter at 500–530 nm. The images were processed using the NIS-Element Software (Nikon).

### Total RNA extraction and cDNA preparation

Frozen tissues were ground in liquid nitrogen and total RNA was extracted with the RNeasy Plant Mini Kit (Qiagen, Courtaboeuf, France) according to the manufacturer's instructions. Extracts were treated with DNase I (0.02 U μL^−1^, New England Biolabs, Ipswich, MA, USA). The total RNA integrity was checked by electrophoresis on 1% (w/v) agarose gels. Using oligo(dT)20 as a primer, cDNA was prepared from samples (0.5 μg) of total RNA using the Superscript II Reverse Transcriptase kit (Invitrogen, Carlsbad, CA, USA).

### Molecular cloning of *P. ramosa* cDNAS

The cDNAs isolated from flowering shoot (stage V) were used for PCR amplification. Degenerate primers corresponding to highly conserved SUT regions were designed (Table 1). After denaturation at 94°C for 5 min, the amplification consisted of 35 cycles of 45 s at 94°C, 45 s at 55°C. An additional final step of elongation was done at 72°C for 5 min. The amplified DNA fragments were purified and cloned into pGEM-T Easy vector (Promega, Madison, WI, USA). Recombinant plasmid DNA were prepared and then sequenced by GATC Biotech (Konstanz, Germany). Based on the initial *PrSUT* sequence informations, new primers were generated for RACE of each fragment using the Generacer kit (Invitrogen). The RACE products corresponding to SUT-encoding genes were cloned and sequenced. To amplify full-length *PrSUT*s, specific primer pairs were designed (Table 1).

**Table 1:** **Primers used in the present study**.

**Gene**	**Primer**	**Forward and reverse primers 5′ → 3′**	**size (bp)**	**Application**
*PrSUT1*	SUT-fwd-deg	TWHATHTGGYTNTGYGGNCC	718	Partial cDNA cloning
*PrSUT2*	SUT-rev-deg	TCNYKNSCCATCCARTCDGTRTC	898	
*PrSUT3*			709	
*PrSUT1*	FLfwd-Pr-SUT1	ACACCTACTACTCTCTACACTCCTATGCTT	1787	Full-length cDNA cloning
	FLrev-Pr-SUT1	ATGCAATCCCACAATATCATATAGTATTC		
*PrSUT2*	FLfwd-Pr-SUT2	TTTTCACATAGAATTATGGATGCAGATTCG	2176	
	FLrev-Pr-SUT2	GATCTTTCCCCAACTATTATTCAAAATCAG		
*PrSUT3*	FLfwd-Pr-SUT3	TAGTCAAGAAATAAGATTAGTTAGATATACTGA	1662	
	FLrev-Pr-SUT3	AACCAACAAGTACTAGTTTATTTAGGTAGTCTC		
*PrEF1α1*	Q-Pr-EF1-UP	TTGCCGTGAAGGATCTGAAAC	63	RT-PCR
	Q-Pr-EF1-DOWN	CCTTGGCAGGGTCGTCTTTA		
*PrSUT1*	Q-Pr-SUT1-UP	CGGGTTCATACACCCACCTCTA	66	
	Q-Pr-SUT1-DOWN	AGTATATGTCGCAGGCTGTGGTT		
*PrSUT2*	Q-Pr-SUT2-UP	GCATTTGGTTTGCTATTGAATTCTG	67	
	Q-Pr-SUT2-DOWN	GGCACATTGGCTCAATAAAGAAG		
*PrSUT3*	Q-Pr-SUT3-UP	CCTTCGGTGCGGCTCTT	54	
	Q-Pr-SUT3-DOWN	CAGCGGCATAACCAATCAAA		
*PrSUT1*	Pr-SUT1-GaWafwd	CACCATGGAGGCCGGTGATAATCTG	1513	CDS cloning for fusion with fluorescent dyes
	Pr-SUT1-GaWarev	ATGAAATCCTCCCATAGTCATAGCCTTG		
*PrSUT3*	Pr-SUT3-GaWafwd	CACCATGGGTAATTCGGACATTATGGA	1495	
	Pr-SUT3-GaWarev	GTGAAATCCTCCGACAGCCAAAGGCTT		
*AtSUC2*	AtSUC2-GaWafwd	CACCATGGTCAGCCATCCAATGGAGAAAG	1540	
	AtSUC2-GaWarev	ATGAAATCCCATAGTAGCTTTGAAGGCAGGA		
*AtKCO5*	AtKCO5-GaWafwd	CACCATGGAACCACTCATCAGCCCACA	1228	
	AtKCO5-GaWarev	CAAAGGATCCCCCAAAAGATCAGGTA		

### Real time RT-PCR

The determined sequences were used to design gene-specific primers for real time RT-PCR (RT-PCR). Six primer pairs were designed (maximum length, 150 bp; optimal melting temperature T_m_ at 60°C; GC percentage between 30 and 80%) using Primer Express 3.0 software (Applied Biosystems, Carlsbad, CA, USA) (Table 1). The selected primers underwent an extensive search using the BLAST tool to avoid any significant homology with other known nucleotide sequences.

A RT-PCR using SYBR Green technology was carried out on an Applied Biosystems 7300 real-time PCR system, as previously described by Péron et al. (2012). All RT-PCR runs contained negative controls with no cDNA template. The amplicon of the constitutive elongation factor *PrEF1*α (Table 1), which showed low cycle threshold variation (SD <0.5 cycle threshold), was used as an internal control to normalize all the data. Three biological replicates were performed, each in three technical replicates. An analysis of variance (ANOVA) was performed on the results from RT-PCR analyses using SigmaPlot version 10.0 (*P* < 0.05, SNK test).

### *In situ* hybridization experiments

The digoxigenin (DIG)-labeled RNA probes were prepared using digoxigenin-11-*UTP* (Roche Diagnostics, Meylan, France) and an *in vitro* transcription kit (Riboprobe Combination Systems, Promega) according to the manufacturer's instructions. The riboprobes were synthesized from the full-length *PrSUTs* clones. The antisense and sense probes were transcribed from SP6 or T7 RNA polymerase promoters after linearization of the vector with *Apa*I or *Nde*I, respectively. The full-length probes were treated by alkaline hydrolysis, as described previously by Péron et al. (2012), thus producing 250 bp fragments. The *in situ* hybridization methods used for *PrSUT*s transcript localization on the parasite shoot sections (10 μm thick) were performed as previously described in Péron et al. (2012).

### Transient expression into *arabidopsis thaliana* protoplasts

The PrSUTs corresponding coding sequences (CDS) were fused with the red fluorescent protein (RFP), in the vectors designed for transient expression in *A. thaliana* leaf protoplasts. The same procedure was used to obtain vectors with CDS of AtSUC2 (accession number: At1g22710) and AtKCO5 (accession number: At4g01840) fused with the green fluorescent protein (GFP). AtSUC2-GFP and AtKCO5-GFP were used in coexpression experiments to validate plasmalemma and tonoplast localization, respectively (Sauer and Stolz, 1994; Voelker et al., 2006). The Phusion proofreading polymerase (Thermo Fisher Scientific, Waltham, MA, USA) was used for amplification following the PCR conditions recommended by the manufacturer. The different primers used for each coding sequence amplification are listed in Table 1. The same procedure as Candat et al. (2013) was used to make the fluorescent dyes-tagged proteins (FDP) constructs. The PCR products corresponding to the full coding sequence (without stop codon) were cloned first in the pENTR/D-TOPO cloning vector (Thermo Fisher Scientific, Waltham, MA, USA) and then recombined using the LR clonase II kit (Thermo Fisher Scientific, Waltham, MA, USA) into the appropriate expression vector (p2GWR7.0; p2GWF7.0) (Karimi et al., 2002) obtained from Plant System Biology (Ghent University. Ghent, the Netherlands). Each coding sequence was cloned upstream from the RFP or GFP sequence in the expression plasmid to produce a C-terminal FDP. The cloning and expression plasmids were amplified in *Escherichia coli* One Shot DG1 cells (Invitrogen, Carlsbad CA, United States) and purified from an overnight culture using the NucleoSpin plasmid purification kit (Macherey Nagel, Hoerdt, France).

Protoplast isolation from *A. thaliana* leaves, as well as transient expression assays and CLSM analysis of the subcellular FDP localization were conducted as previously described by Candat et al. (2013, 2014). GFP, RFP, and chlorophyll were excited with 488-, 561-, or 638-nm laser lines, respectively, with an emission band of 500 to 550 nm for GFP detection, 570 to 620 nm for RFP detection, and 662–737 nm for chlorophyll autofluorescence.

### Sequence analysis

The bioinformatics analyses were performed using Vector NTI 9.1.0 software (Invitrogen, Invitrogen Corporation, Carlsbad, CA, USA). The sequence homologies were verified against GenBank databases using BLAST programs (http://www.ncbi.nlm.nih.gov/blast). The phylogenetic analyses were conducted with MEGA4 software (Tamura and Akutsu, 2007). The neighbor-joining consensus tree was inferred from 500 bootstrap replicates. The amino acid sequences were aligned with Clustal W. Structural insights about PrSUT proteins were obtained using PSIPRED application (Protein Structure Prediction Server, http://bioinf.cs.ucl.ac.uk/psipred/). The subcellular targeting predictions were performed using the freely available online program: WoLF PSORT (http://wolfpsort.org/) (Horton et al., 2007), which is used as a general subcellular localization predictor using the plant data sets.

## Results

### Phloem unloading pathways in tubercle and shoot

**Figure 1:**
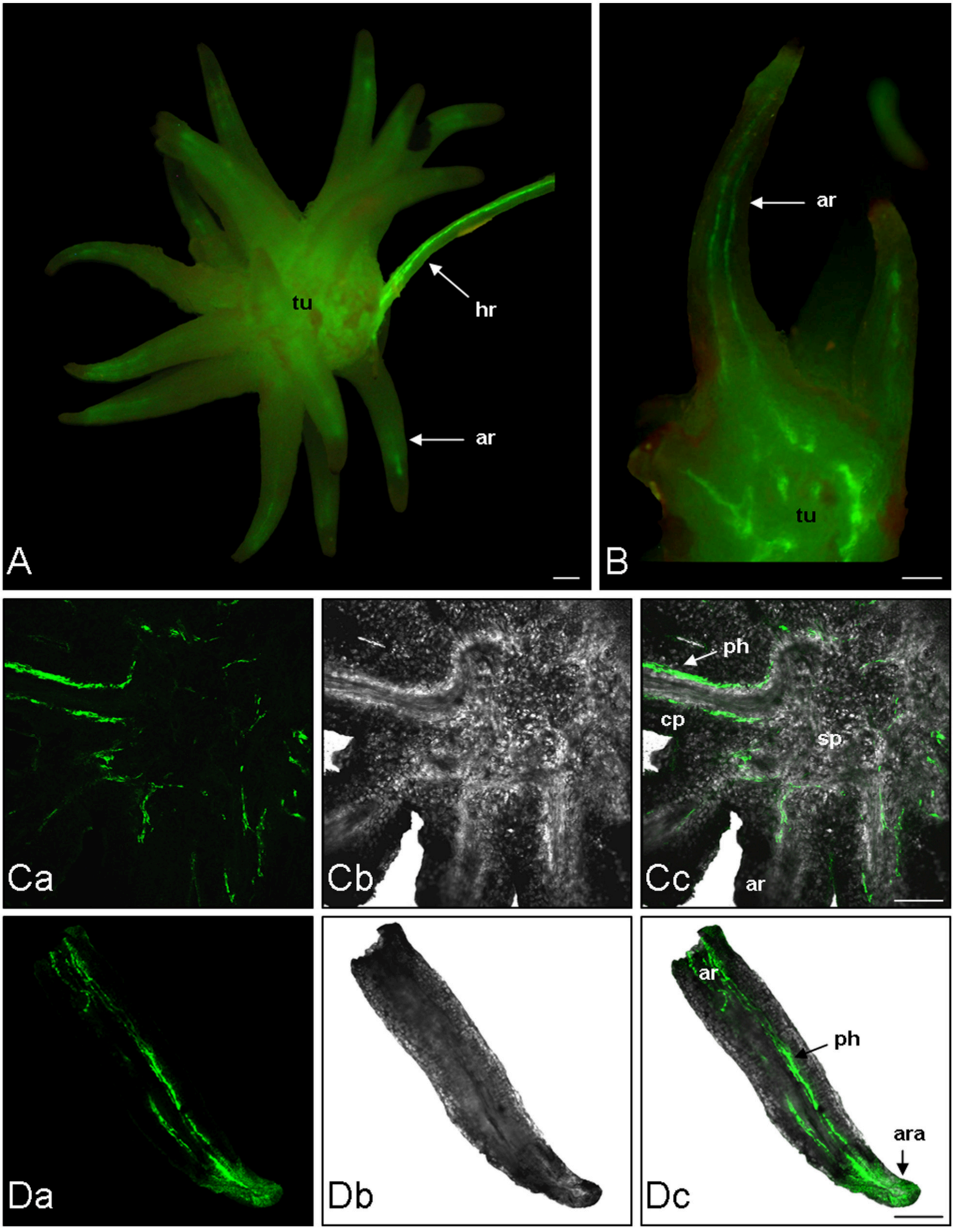
**Analysis of phloem unloading into *P*. *ramosa* parasite using fluorescent dye**. CFDA was loaded on abraded mature leave of host plant. After 2 h CF translocation from host to parasite (tubercle) was monitored by fluorescence microscopy and CLSM. **(A)** Fluorescence image of a *Brassica napus* (cv. Campo) host root bearing a *P. ramosa* tubercle. **(B)** Fluorescence image of a free hand cross section of the same tubercle. **(Ca)** CLSM image of a vibratome cross-section (200 μm thick) of tubercle. **(Cb)** Transmitted light picture of the same region. **(Cc)** Computer-assisted overlay of the CLSM and the transmitted light picture. **(Da)** CLSM image of a vibratome longitudinal-section (200 μm thick) of an adventitious root of tubercle. **(Db)** Transmitted light picture of the same region. **(Dc)** Computer-assisted overlay of the CLSM and the transmitted light picture. ar, adventitious root; ara, adventitious root apex; hr, host root; ph, phloem; sp, storage parenchyma; tu, tubercle. Green, GF signal. Bars: 500 μm.

**Figure 2:**
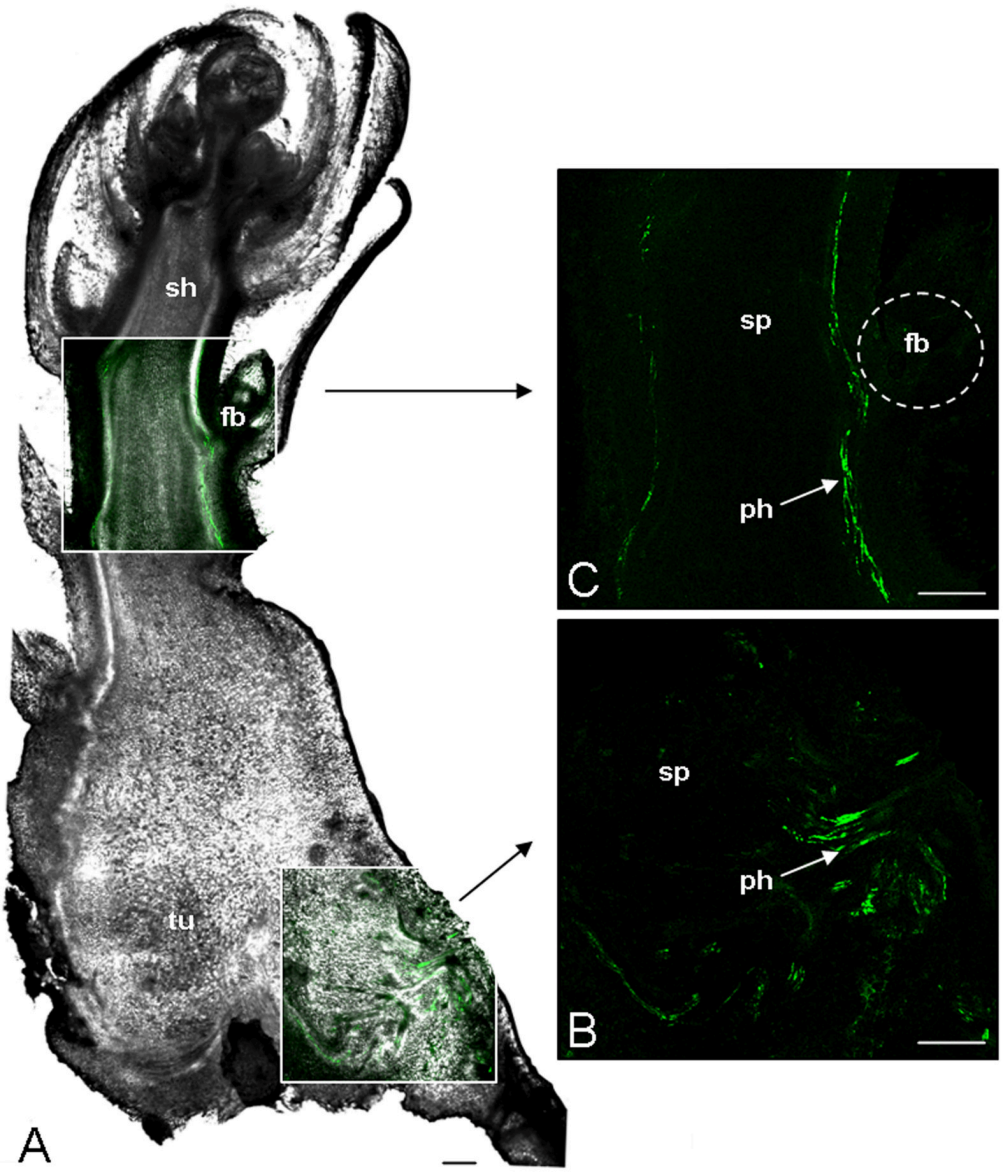
**Analysis of phloem unloading into *P. ramosa* parasite using fluorescent dye**. CFDA was loaded on abraded mature leave of host plant. After 2 h CF translocation from host to parasitic tissues was monitored by CLSM. **(A)** Transmitted light picture of a vibratome longitudinal-section (200 μm thick) of parasitic tubercle bearing the growing subterranean shoot. The two white boxed areas, one in tubercle another in shoot, are computer-assisted overlay of the CLSM and the transmitted light pictures. **(B)** CLSM image of the tubercle boxed area. **(C)** CLSM image of the shoot boxed area. fb, floral bud; ph, phloem; sh, subterranean shoot; sp, storage parenchyma; tu, parasitic tubercle. Green, GF signal. Bars: 500 μm.

The phloem continuity between the host and the parasite was analyzed by using carboxyfluorescein (CF) as a symplastic tracer which is produced from CFDA by plant esterase activities. Two hundred microliter of CFDA solution was applied on an abraded mature leaf of the host plant (*Brassica napus*). The CF confinement in leaf phloem was confirmed by the observation of fluorescence in the leaf veins 1 h after CFDA treatment (data not shown). Although autofluorescence was not detected in the host root and the tubercle without CFDA treatment, both organs exhibited a bright green fluorescence 2 h after CFDA treatment, indicating that CF was translocated from the host root to the tubercle through symplastic phloem connections (Figure 1A). The CF fluorescence signal was relatively diffuse in the tubercle but still appeared stronger in the conducting tissues, notably in the adventitious roots (Figure 1B). According to the CLSM images, CF was restricted to the phloem tissues inside the tubercle, showing the symplastic isolation of the phloem tissues from the storage parenchyma (Figures 1Ca–Cc). In contrast, the CF release in the cortex was observed at the apex of the adventitious roots (Figures 1Da–Dc). When CFDA was applied on a host leaf later during the subterranean development of the parasite, CF accumulation in the parasitic plant was restricted to the phloem tissues in the tubercle as well in the growing shoot, the adventitious roots being senescent at this developmental stage (Figure 2). A CF-related fluorescence was not observed in the shoot apex. In addition, the dormant floral buds appeared symplastically isolated from the shoot phloem tissues. All these results demonstrate that the apoplastic phloem unloading prevails in multiple sink areas during growth of the parasitic plant, except in the apices of adventitious roots, and then underline the critical role of sucrose transporters in sucrose phloem unloading in the parasitic plant.

### Cloning and characterization of three *PrSUT* sucrose transporter genes

**Figure 3:**
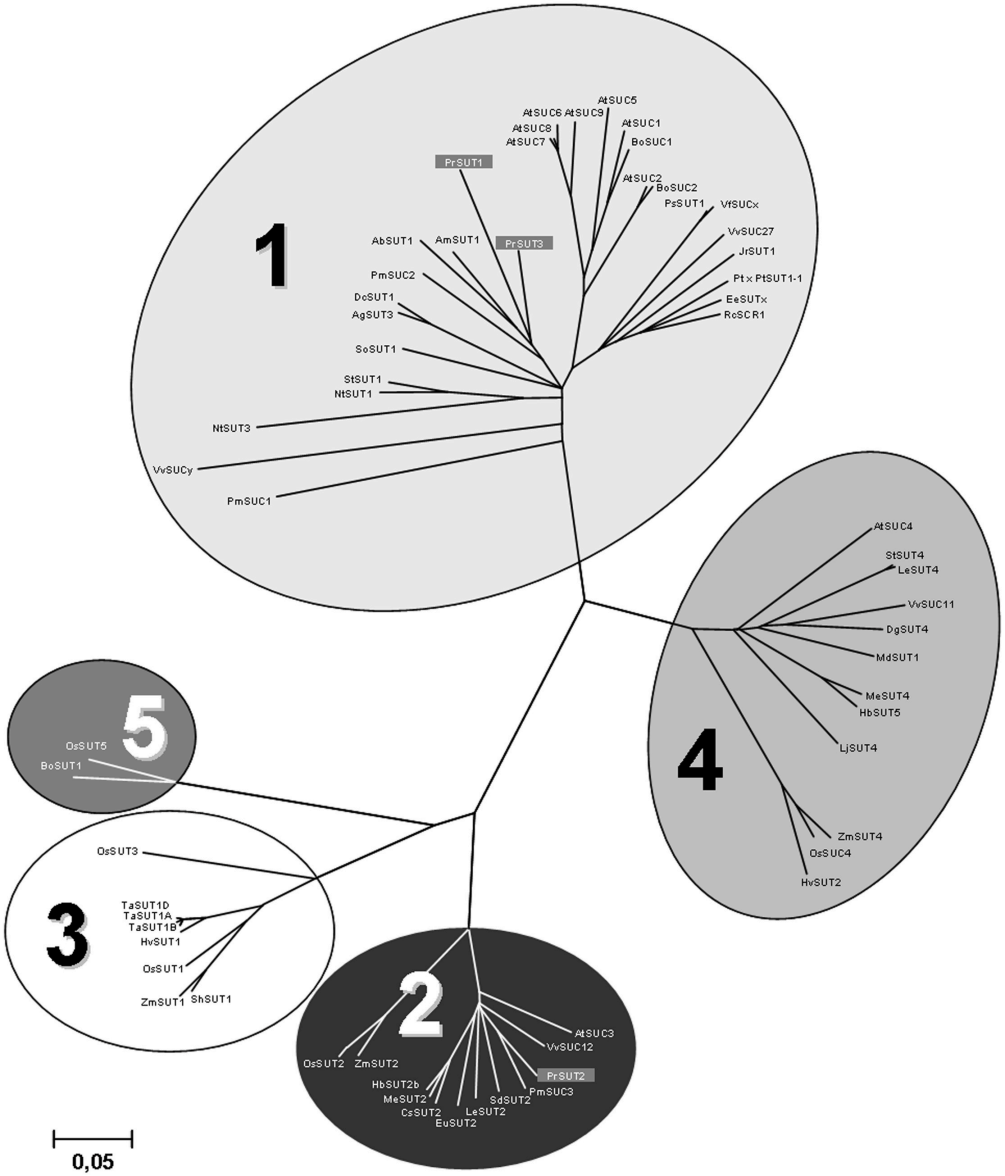
**Phylogenetic tree for confirmed or predicted sucrose transporter sequences from publicly accessible databases**. Complete *Phelipanche ramosa* sucrose transporter predicted protein sequences were compared to sucrose transporters from other plant species using ClustalW. Five phylogenetic Groups defined by Kühn and Grof (2010) are indicated. Accession numbers of presented sucrose transporter sequences are: AbSUT1 (*Asarina barclaiana*; AAF04294), AgSUT3 (*Apium graveolens*; ABB89051), AmSUT1 (*Alonsoa meridionalis*; AAF04295), AtSUC1 (*Arabidopsis thaliana*; At1g71880), AtSUC2 (At1g22710), AtSUC3 (At2g02860), AtSUC4 (At1g09960), AtSUC5 (At1g71890), AtSUC6 (At5g43610), AtSUC7 (At1g66570), AtSUC8 (At2g14670), AtSUC9 (At5g06170), BoSUC1 (*Brassica oleracea*; AAL58071), BoSUC2 (*B*. *oleracea*; AAL58072), BoSUT1 (*Bambusa oldhamii*; AAY43226), CsSUT2 (*Citrus sinensis*; AAM29153), DgSUT4 (*Datisca glomerata*; CAG70682), DcSUT1 (*Daucus carota*; BAA89458), EeSUCx (*Euphorbia esula*; AAF65765), EuSUT2 (*Eucommia ulmoides*; AAX49396), HbSUT2b (*Hevea brasiliensis*; ABJ51932), HbSUT5 (*H*. *brasiliensis*; ABK60189), HvSUT1 (*Hordeum vulgare*; CAB75882), HvSUT2 (*H*. *vulgare*; CAB75881), JrSUT1 (*Juglans regia*; AAU11810), LjSUT4 (*Lotus japonicus*; CAD61275), MdSUT1 (*Malus* × *domestica*; AAR17700), MeSUT2 (*Manihot esculenta*; ABA08445), MeSUT4 (*M*. *esculenta*; ABA08443), NtSUT1 (*Nicotiana tabacum*; X82276), NtSUT3 (*N*. *tabacum*; AAD34610), OsSUT1 (*Oryza sativa*; AAF90181), OsSUT2 (*O*. *sativa*; AAN15219), OsSUT3 (*O*. *sativa*; BAB68368), OsSUC4 (*O*. *sativa*; Q2QLI1), OsSUT5 (*O*. *sativa*; BAC67165), PmSUC1 (*Plantago major*; CAI59556), PmSUC2 (*P*. *major*; X75764), PmSUC3 (*P*. *major*; CAD58887), PrSUT1 (*Phelipanche ramosa*; ALI88692), PrSUT2 (*P*. *ramosa*; ALI88693), PrSUT3 (*P*. *ramosa*; ALI88694), Pt × PtSUT1-1 (*Populus tremula* × *Populus tremuloides*; CAJ33718), PsSUT1 (*Pisum sativum*; AAD41024), RcSCR1 (*Ricinus communis*; CAA83436), SdSUT2 (*Solanum demissum*; AAT40489), SlSUT2 (*Solanum lycopersicum*; AAG12987), SlSUT4 (*S*. *Lycopersicum*; AAG09270), ShSUT1 (*Saccharum hybridum*; AAV41028), SoSUT1 (*Spinacea oleracea*; Q03411), StSUT1 (*Solanum tuberosum*; CAA48915), StSUT4 (*S*. *tuberosum*; AAG25923), TaSUT1A (*Triticum aestivum*; AAM13408), TaSUT1B (*T*. *aestivum*; AAM13409), TaSUT1D (*T*. *aestivum*; AAM13410), VfSUCx (*Vicia faba*; CAB07811), VvSUCy (*Vitis vinifera*; AAL32020), VvSUC11 (*V*. *vinifera*; AAF08329), VvSUC12 (*V*. *vinifera*; AAF08330), VvSUC27 (*V*. *vinifera*; AAF08331), ZmSUT1 (*Zea mays*; BAA83501), ZmSUT2 (*Z*. *mays*; AAS91375), ZmSUT4 (*Z*. *mays*; AAT51689). The neighbor-joining consensus tree was inferred from 500 bootstrap replicates. Bar indicates the evolutionary distance.

Three partial *P*. *ramosa* cDNAs were cloned using sets of primers designed from conserved regions of plant sucrose transporter sequences. Using RACE strategies, three full-length cDNAs: *PrSUT1, PrSUT2*, and *PrSUT3* (accession numbers: KR559018, KR559019 and KR559020, respectively) were isolated. The *PrSUT1, PrSUT2*, and *PrSUT3* sequences encode 503, 605 and 497 amino-acid proteins, with predicted molecular masses of 52.5, 65.3, and 52.3 kDa, respectively. The PrSUT1 and PrSUT3 amino acid sequences share the highest levels of identity with AmSUT1 sequence (*Alonsoa meridionalis*, AAF04295; 76.6 and 68%, respectively) (Knop et al., 2001), whereas the PrSUT2 amino acid sequence shares high identity with the PmSUC3 sequence (*Plantago major*, CAD58887; 78.7%) (Barth et al., 2003). The analysis of the deduced amino acid sequences of the putative *PrSUT* genes, using PSIPRED application (Protein Structure Prediction Server, http://bioinf.cs.ucl.ac.uk/psipred), revealed that all three SUTs contained 12 transmembrane domains with both N- and C-termini on the cytoplasmic side (data not shown), as often found in other sugar and notably sucrose transporters (Lalonde et al., 2004). The PrSUT2 sequence differs from others PrSUT sequences by an N-terminal extension and a central cytoplasmic loop (between transmembrane segments VI and VII) (Supplemental material 1). A phylogenetic analysis of SUT orthologs, based on the five phylogenetic groups defined by Kühn and Grof (2010) and including the PrSUT predicted proteins was realized (Figure 3). Groups 3 and 5 represent the monocot-specific branches and group 1 represents the dicot-specific branch. PrSUT1 and PrSUT3 belonged to the group 1, whereas PrSUT2 falls into the group 2. Groups 2 and 4 are both composed of monocot- and dicot-specific subclades.

### Development-related changes in *PrSUT* transcript accumulation

**Figure 4:**
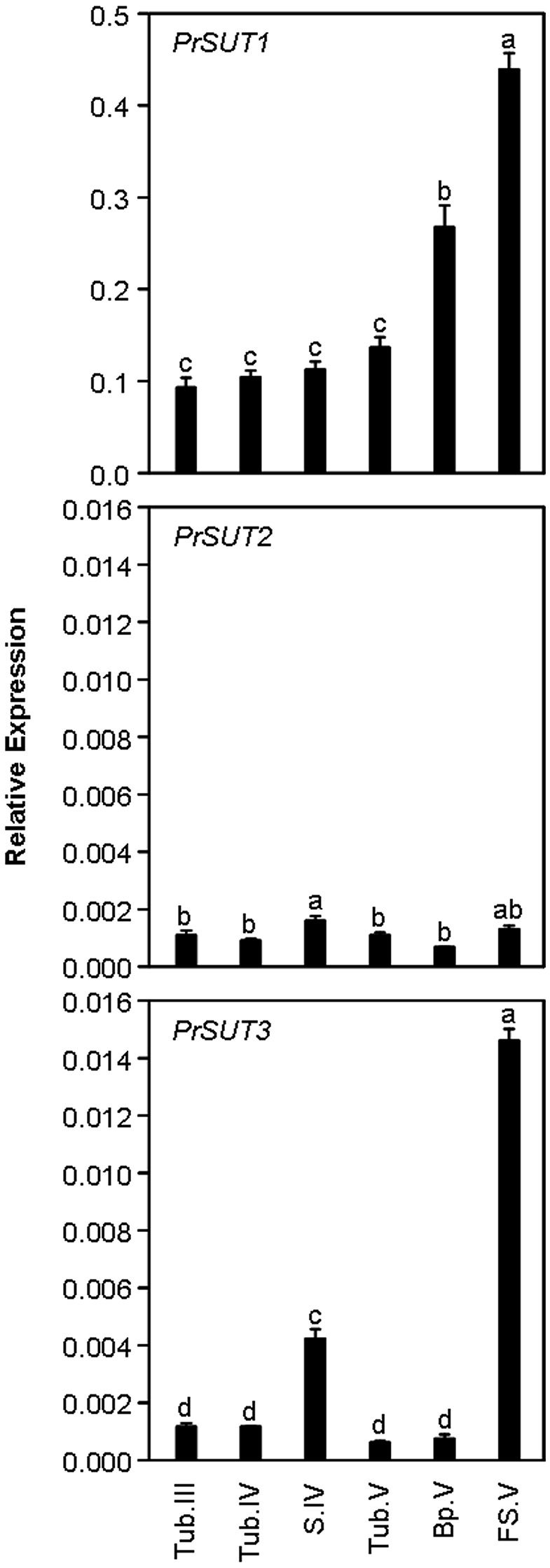
**Development-related changes in the levels of *PrSUT* transcripts in *Phelipanche ramosa***. *PrSUT* transcripts accumulation is expressed relative to *EF1*-α*1* transcript levels. Data are means ± SE (*n* = 3). Values with the same letter are not significantly different (ANOVA, SNK test, *P* < 0.05). Developmental stages are parasitic stages following attachment to host roots: growing tubercle (Tub.III); tubercle (Tub.IV) bearing the growing subterranean shoot (S.IV); tubercle (Tub.V) bearing the flowering shoot (following emergence); growing flowering shoot (apical part) (FS.V). Basal part (Bp.V) does not bear flowers and is larger and more fibrous than the flowering shoot (see Draie et al., 2011 for photographs).

While *PrSUT1* had a constant transcript accumulation level in tubercle throughout the complete parasite development, *PrSUT1* mRNA accumulated to a two-three fold higher extent in both basal and apical parts of the growing shoot once emerged from the soil (Bp.V, FS.V) (Figure 4). In contrast, *PrSUT2* transcripts were constantly accumulated at low levels in both tubercle and shoot (Figure 4). *PrSUT3* mRNA was preferentially accumulated in the shoot, primarily during post-emergence growth. *PrSUT3* transcripts were four-fold more abundant in the apical (growing) part than in the basal part of the emerged shoot.

### Tissue-specific expression of *PrSUT1* and *PrSUT3* and subcellular localization of the respective proteins

**Figure 5:**
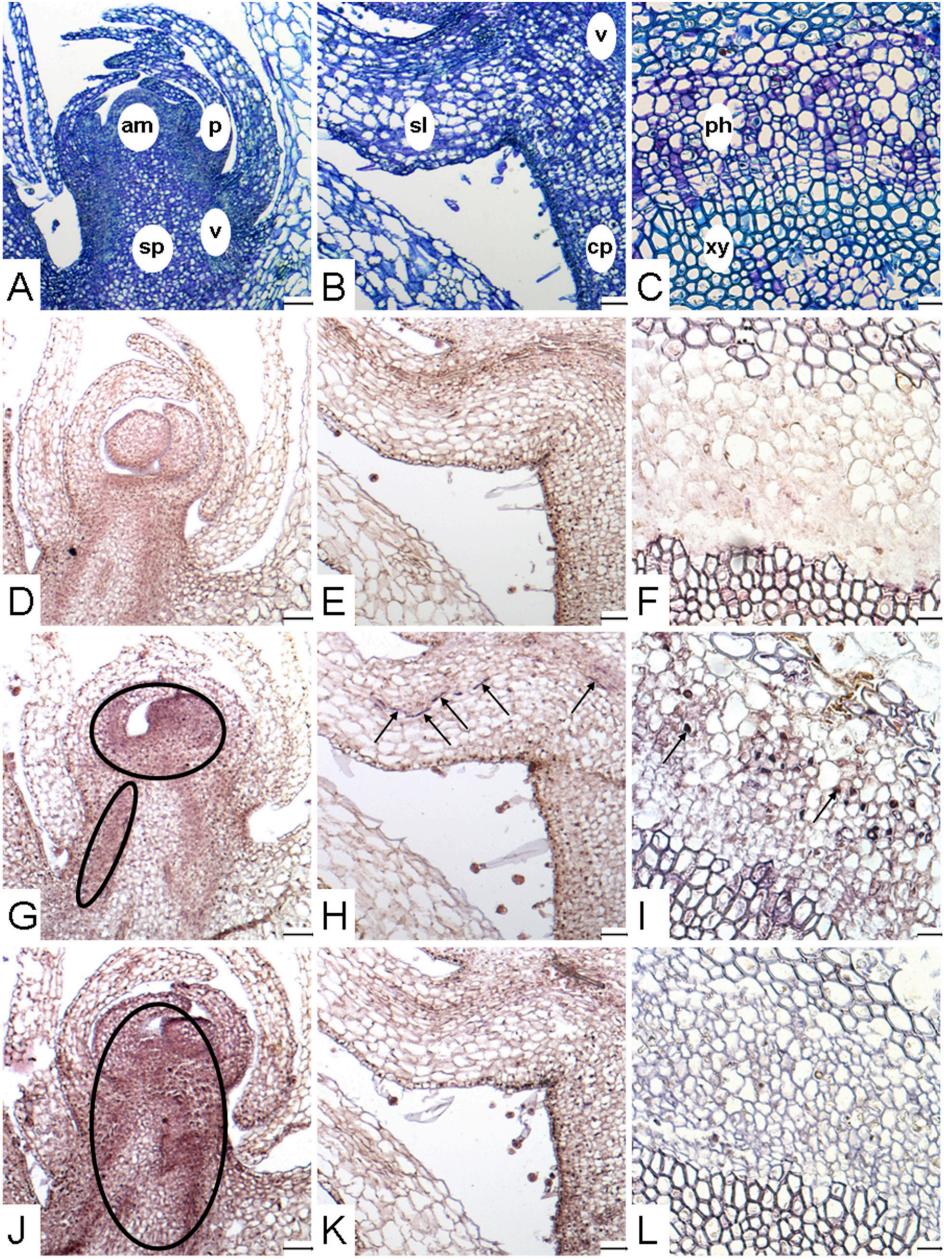
**Localization of *PrSUT* expression in *P*. *ramosa*. (A–L)** Paraffin-embedded sections (10 μm thick) of parasitic tissues. **(A,D,G,J)** Longitudinal sections focused on the apical part of growing subterranean shoot (stage S.IV). **(B,E,H,K)** Longitudinal sections focused on a scale leaf in the basal part of subterranean shoots (stage S.IV). **(C,F,I,L)** Cross-sections focused on mature vascular tissues in the apical part of flowering shoots (stage FS.V). **(A–C)** Sections stained with TBO, showing the different tissues of shoot. **(D–F)** Section showing *in situ* hybridization signal obtained with *PrSUT* sense probes. The sense probe picture is representative for both probes. **(G–I)** Section showing *in situ* hybridization signal obtained with *PrSUT1* antisense probe. **(J–L)** Section showing *in situ* hybridization signal obtained with *PrSUT3* antisense probe. Open ovals and black arrows indicate positive hybridization signals. am, apical meristem; cp, cortical parenchyma; p, scale-leaf primordium; ph, phloem; sl, scale leave; sp, storage parenchyma; v, vasculature (immature vascular tissues); xy, xylem. Bars: **(A,B,D,E,G,H,J,K)**: 100 μm; **(C,F,I,L)**: 25 μm.

Because *PrSUT2* expression was low throughout the parasite development, the analyses of tissue-specific expression of transcripts and subcellular localization of the respective proteins were only conducted for *PrSUT1* and *PrSUT3*. Based on *PrSUT* gene-expression profiles, both subterranean and flowering shoots (S.IV, FS.V) were chosen for *in situ* hybridization experiments (Figure 5). Tissue organization was outlined with Toluidine blue O (TBO) staining of sections (Figures 5A–C) which highlighted young vascular tissue (vasculature) in stem and scale leaf and multiple sink areas including shoot apical meristem, scale leaf primordia, large parenchyma cells close to vascular tissue in stem (storage parenchyma) and scale leaves. Positive hybridization (purple stain) with the specific antisense probes revealed *PrSUT1* (Figures 5G–I) and *PrSUT3* (Figures 5J–L) transcript accumulation, whereas no significant signal was observed after hybridization with sense probes (Figures 5D–F). A weak staining suggests *PrSUT1* transcript accumulation in the apical sink tissues (Figure 5G), including meristem, scale-leaf primordia and likely immature vascular tissues (vasculature, diffuse staining). More evident accumulation in the vascular tissues was detected especially in the mature phloem cells of subterranean (basal part, scale leaves) and flowering (apical part) shoots (Figures 5H,I). No *PrSUT1*-related staining was observed in the storage parenchyma (Figures 5G,H). In the meantime, an evident *PrSUT3* transcripts accumulation was detected in multiple apical sink tissues including apical meristem, leaf-scale primordia, vasculature and even storage parenchyma (Figure 5J), but no transcripts were found in the mature vascular tissues (Figures 5K,L).

**Figure 6:**
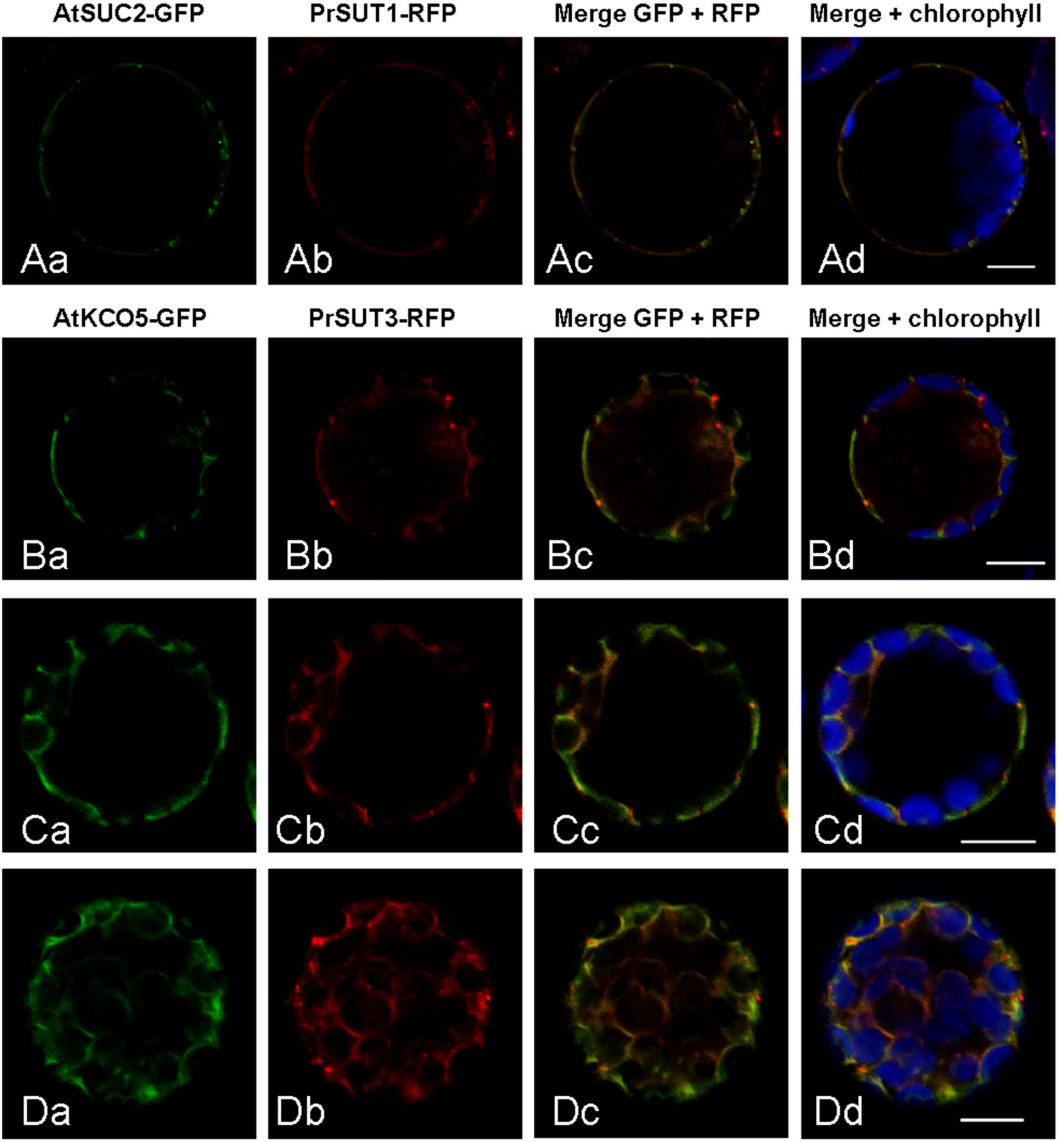
**Subcellular localization of PrSUT proteins using transient expression of translational fluorescent proteins fusions in *Arabidopsis* mesophyll protoplasts. (Aa–d)** PrSUT1-RFP fusion protein was co-expressed with an *Arabidopsis* plasmalemma protein (AtSUC2) fused to GFP. **(Ba–d, Ca–d, Da,d**) PrSUT3-RFP fusion protein was co-expressed with an *Arabidopsis* tonoplast protein (AtKCO5) fused to GFP. Green, GFP; red, RFP; blue, chlorophyll. Bars: 10 μm.

As sucrose transporters of the group 1 family, PrSUT1 and PrSUT3 are expected to reside in the plasma membrane. However, an *in silico* analysis with the sub-cellular predictive software WoLF PSORT (http://wolfpsort.org) (Horton et al., 2007) suggests that PrSUT3 could be a tonoplast protein, since among the list of 14 k-nearest neighbors for PrSUT3 analysis, 11 proteins are tonoplastic. In the case of PrSUT1, the PWolfSort analysis concluded to a plasma membrane localization (8/14 proteins), but five others proteins in the k-nearest neighbors list were localized in the tonoplast. To go beyond these predictions, we investigated the subcellular localization of PrSUT1 and PrSUT3 using transient expression of proteins tagged with fluorescent proteins into *Arabidopsis* protoplasts (Figure 6). The results revealed a clear fluorescence co-localization of PrSUT1-RFP with that of AtSUC2-GFP, a plasma membrane protein (Figures 6Aa–d). Both fusion proteins were systematically detected in a thin layer at the periphery of protoplasts, with additional spots that could possibly correspond to Golgi vesicles in which the proteins would accumulate during their routing toward plasma membrane. In the case of PrSUT3-RFP, which was co-expressed with AtKCO5-GFP, a tonoplastic protein, the pattern of expression of the fusion was different from that of PrSUT1-RFP/AtSUC2-GFP (Figure 6). Both the fact that the fusion proteins were largely co-localized, and that fluorescence was peripheral in the regions without large organelles, such as chloroplasts, and delineated the internal side of chloroplasts and cytoplasmic strands, argue in favor of a tonoplast localization (Figures 6Ba–d; Ca–d; Da–d). However, there are also regions with differential accumulation of the two fusion proteins (e.g., punctae of PrSUT3-GFP), and others where the fusion proteins can be observed on the external part of chloroplasts (e.g., Figure 6Cd), which would not fit with a tonoplast localization. In such experiments using an artificial system, one cannot rule out a possible mis-targeting of the proteins. Although more experiments would be needed to definitely ascertain the localization of PrSUT3, these experiments strongly suggest that this protein is targeted to the tonoplast, while PrSUT1 would be a plasma membrane protein, as expected.

## Discussion

### CFDA application to host plant traces phloem continuity in the whole host-parasite interaction

The host plant-parasitic plant interaction represents a distinctive model in terms of source-sink relationships in plants, in which the haustorium is a decisive component which connects host source to parasite sink. Several studies focusing on the haustorium structure in *Orobanchaceae* plants and herbicide or macromolecule trafficking at the host-parasite interface demonstrated that host and parasite phloem tissues were connected symplastically through functional interspecific plasmodesmata within the haustorium (Westwood et al., 2009; Aly et al., 2011; Smith et al., 2013). This was confirmed in the present study concerning the parasitic plant species *P*. *ramosa* parasitizing *B. napus* through the use of the symplastic tracer CF which was previously used for analysing the phloem unloading pathways in plants (Zhang et al., 2006; Bederska et al., 2012; Wang et al., 2015). CFDA experiments here provided evidence for the symplastic phloem continuity from host leaves to both tubercle and shoot of the parasitic plant, hence explaining how the latter can divert resources from the host plant, especially sucrose, to support parasite growth driven by high sugar accumulation in vegetative sinks (Aber et al., 1983; Delavault et al., 2002; Draie et al., 2011). The pathways of sucrose phloem unloading and storage in tubercle and shoot take an important place in the host source–parasite sink relationships, which are clarified in the present study.

### The apoplastic phloem unloading pathway dominates in both tubercle and shoot, but not in the adventitious roots

In the parasitic plant, the CFDA experiments revealed that phloem tissues were not directly connected to most of the sink tissues in both tubercle and shoot, but with the exception of the apical root apices where phloem was shown to connect symplastically. Interestingly, this scenario differs drastically from that of a multitude of plants in which the symplastic pathway predominates for phloem unloading in root and shoots apices as well as in vegetative storage sinks, such as stems, roots and tubers (Lemoine et al., 2013). Since the sink strength of the parasitic plant relies primarily on a large sugar accumulation in tubercle and shoot, the apoplastic unloading in both vegetative organs should be interpreted as a relevant strategy to keep phloem unloading and parasite growth despite the consecutive enrichment in sugars, thus preventing symplastic backflow. Similarly, such a strategy was shown to occur in growing fruits which accumulate large amounts of sugars (Jin et al., 2009; Lemoine et al., 2013). Concerning the symplastic phloem unloading in the adventitious root apices, this scenario should support transient rapid growth of these organs, resulting in a particular phenotype of tubercles called “spider” and acting as a major component of the sink strength of the parasitic plant during early developmental stages. Later, as evidenced by the CFDA experiments, the growing shoot calls up the host-derived phloem sap when the adventitious roots of tubercles senesce, and then highlighting change in the dominant sinks during parasite development.

### The sucrose transporter (*PrSUT*) gene family in *P. ramosa*

It was previously shown that symplastic invertase activities, primarily the assumed vacuolar PrSAI1 activity, predominate by far over the apoplastic invertase activity for sucrose utilization in both tubercle and shoot of *P. ramosa* (Draie et al., 2011). These data together with the present finding about the apoplastic unloading in both organs tend to highlight an important role of sucrose carriers, especially SUT proteins, as primary actors in phloem unloading and sugar accumulation in sinks of the parasitic plant.

Three putative SUT-encoding cDNAs were isolated in *P. ramosa*, suggesting that, like in most plants, sucrose transporters are encoded by a small multigenic family in this parasitic species (Sauer, 2007; Kühn and Grof, 2010). The *in silico* analysis showed that the corresponding proteins display the plant SUT common structural features previously described by Lalonde et al. (2004), such as cytoplasmic N- and C-terminal extensions, 12 predicted transmembrane domains and the central cytoplasmic loop between transmembrane segments VI and VII.

The phylogenetic analysis revealed that both PrSUT1 and PrSUT3 belong to the dicot group 1, containing plasma membrane-localized SUT implied in apoplastic phloem loading, sucrose import into sink cells and are thus essential for normal growth (Hackel et al., 2006; Srivastava et al., 2009). PrSUT2 belongs to group 2 of SUT proteins common to monocot and dicot that specifically display an extension of their N-terminal extremity and their central cytoplasmic loop. Interestingly, sequence information from *Phelipanche aegyptiaca* (a specie closely related to *P. ramosa*) contained in the Parasitic Plant Genome Project (PPGP; http://ppgp.huck.psu.edu) database confirmed the existence of the 3 cDNAs we cloned but also reveals the existence of a fourth partial cDNA (OrAeBC1_18026: 402 pb) encoding a predicted protein that shares 65.5% identity with AtSUC4. An *a posteriori* analysis was carried out in *P*. *ramosa*, showing that, like *PrSUT2* transcripts, the *PrSUT4* transcripts were constantly accumulated at low levels in the parasitic plant (Supplemental material 2).

### The *PrSUT1* gene encodes a plasma membrane-localized protein in phloem and sink cells

The transient expression assays in *A. thaliana* protoplasts support the plasmalemma localization of PrSUT1 which was expected from *in silico* analysis. The results of the *in situ* hybridization experiments which underlined transcript accumulation in mature phloem cells of the shoot (stem and scale leaves), as well as in the shoot apical sinks, such as meristem and scale leaf primordia, were consistent with the *PrSUT1* expression patterns in which the apical part of shoots appeared to be the parasitic plant areas where this transcript is the most abundant. All these findings therefore suggest that PrSUT1 is active in the shoot in both phloem functioning (sucrose retrieval) and sucrose uptake by the apical sinks. This is in concordance with the statement that although SUT1 proteins are in the most part essential for phloem loading in leaves, some members have also been detected in sink organs, notably in the phloem cells but also in cells different from phloem cells (sink cells; Kühn and Grof, 2010; Lemoine et al., 2013). As SWEET facilitators were proposed recently to contribute next to SUTs in phloem unloading in plants (Lemoine et al., 2013; Patil et al., 2015), it would be of interest to clarify their role in sucrose export from phloem cells of sink organs in *P. ramosa*.

### The *PrSUT3* gene may encode a tonoplastic protein in sink cells

The expression pattern highlighted the over-expression of *PrSUT3* in the shoot throughout the parasite growth. Indeed, *PrSUT3* is expressed in the young subterranean shoot (S.IV) and, to a higher extent following emergence, in the apical (growing) part of the flowering shoot (S.V). The *PrSUT3* transcripts accumulated in multiple apical sinks, including meristem, scale-leaf primordia, immature vascular tissues and storage parenchyma. Unlike the *PrSUT1* transcripts, the *PrSUT3* transcripts did not accumulate in the mature phloem cells. The tonoplastic localization of PrSUT3, predicted by *in-silico* analysis, was also supported by the transient expression experiments in *A. thaliana* protoplasts. We therefore propose that PrSUT3 acts as a tonoplastic sucrose transporter in sink cells. Although complementary experiments are necessary to fully ascertain its localization, PrSUT3 would therefore be the first SUT belonging to the group 1 which is localized in the tonoplast. Indeed, according to the localisation of GFP fusions, only few members of the group 4 are to date assigned to the tonoplast, AtSUT4 (Endler et al., 2006), HvSUT2 (Endler et al., 2006), LjSUT4 (Reinders et al., 2008), and PpSUT4 (Zanon et al., 2015).

Sucrose influx from the cytosol to the vacuole occurs against the proton gradient and is energically unfavorable, then implying a role for SUT as proton/sucrose antiporter. However up to date, all SUT have been shown functioning like proton/sucrose symporters. Then the involvement of the assumed tonoplastic PrSUT3 in sucrose influx into the vacuole of sink cells is unlikely. The signification of the changes in the expression profile of *PrSUT3* during shoot growth, notably in intracellular sucrose transport, needs clarification. Its function to release sucrose from vacuoles cannot be excluded.

### The *PrSUT* genes as targets for reverse genetics in *P. ramosa*

Reverse genetics in parasitic plants is effective only in few chlorophyllous parasitic plants to date (Ishida et al., 2011, 2016; Bandaranayake et al., 2012). These plants are indeed able to develop without a host, which facilitates the production of transgenic lines. This is not the case for the achlorophyllous parasitic plants like *P*. *ramosa*. Fernandez-Aparicio et al. (2011) have described an efficient method for transforming *P. aegyptiaca* calli and providing transgenic seedlings by inoculating the roots of a host plant with infectious transgenic calli. Otherwise, the efficiency of trans-silencing strategies based on the genetic transformation of the host plant for producing silencing signals which are targeted against a parasite's gene and then transferred to the parasite thanks to haustorial connections, was demonstrated in some host-parasite interactions (Tomilov et al., 2008; Alakonya et al., 2012; Bandaranayake et al., 2012; Bandaranayake and Yoder, 2013), implying notably *Phelipanche* species (Aly et al., 2009, 2011). Then trans-silencing strategies in addition to reverse genetics and likely gene editing should be conceivable for *P*. *ramosa* in a near future and both *PrSUT1* and *PrSUT3*, in addition to *PrSai1*, will be good candidates for dealing phloem unloading in the parasitic plant in depth.

## Author contributions

TP, AC, CV, and GM contributed to the production of the data. TP, DM, PD, and PS contributed to the analysis of the data. TP, DM, and PS wrote the original manuscript and made revisions. PD and PS contributed to the coordination of the project.

## Funding

This work was supported financially by a Ph.D. fellowship (to TP) and funds from the French Ministry of Education and Research.

### Conflict of interest statement

The authors declare that the research was conducted in the absence of any commercial or financial relationships that could be construed as a potential conflict of interest.
