# Supplementary material for: New Insights into Phloem Unloading and Expression of Sucrose Transporters in Vegetative Sinks of the Parasitic Plant Phelipanche ramosa L. (Pomel)
Source: Front Plant Sci. 2017 Jan 9;7:2048. doi: 10.3389/fpls.2016.02048 (PMC5220101; doi:10.3389/fpls.2016.02048)
Supplement: Supplementary file 1 [file Presentation1.PDF]

Supplementary Material:

New Insights into Phloem Unloading and Expression of Sucrose Transporters in Vegetative Sinks of the Parasitic Plant *Phelipanche ramosa L.* (Pomel)

Thomas Péron, Adrien Candat, Grégory Montiel, Christophe Veronesi, David Macherel, Philippe Delavault and Philippe Simier*
*Correspondence:
Pr. Philippe Simier
philippe.simier@univ-nantes.fr

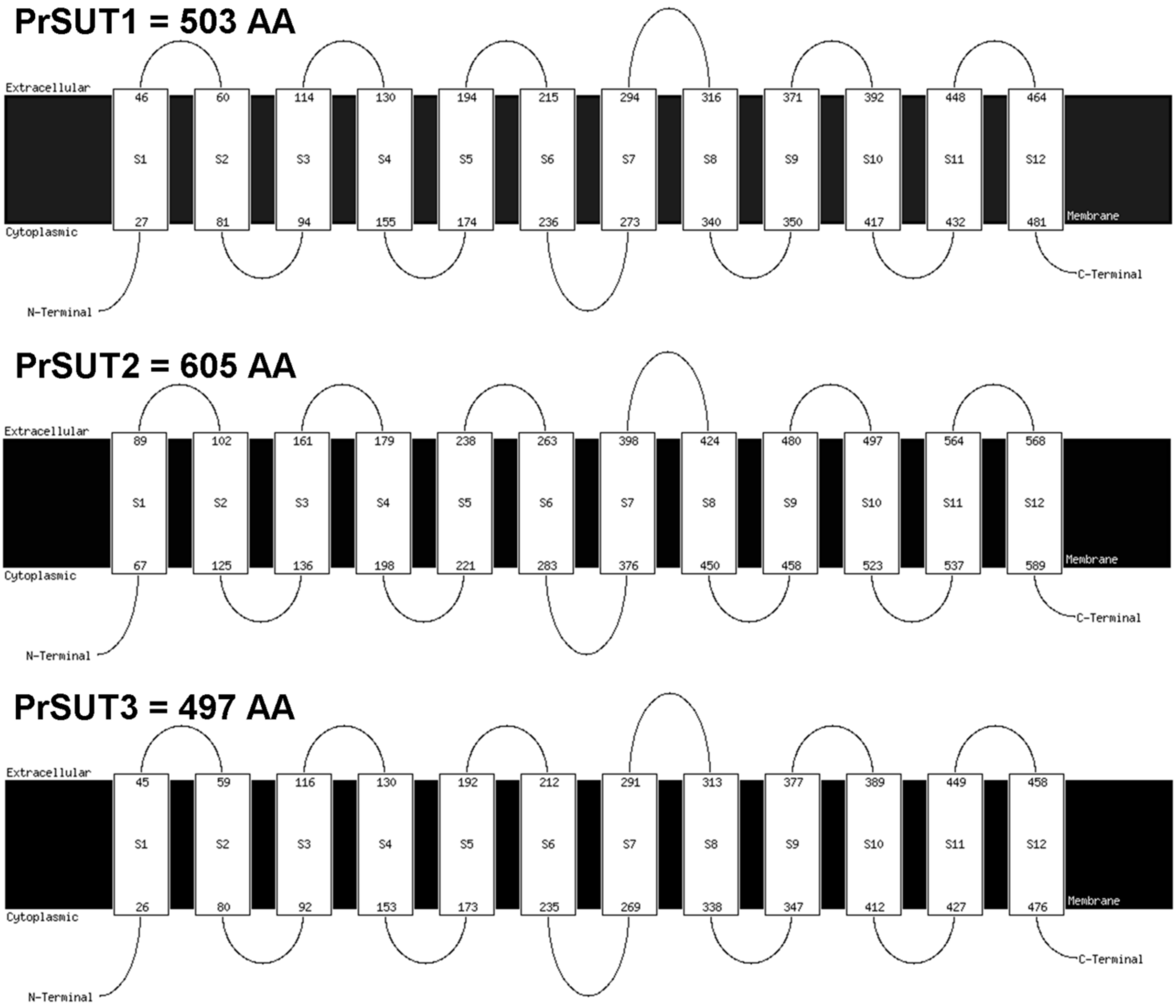

Protein	N-term	Loop S6-S7	C-term
**PrSUT1**	27	37	22
**PrSUT2**	67	93	26
**PrSUT3**	26	34	22

Fig S1. Analysis of the deduced amino acid sequences of the putative *PrSUT* genes using PSIPRED application (Protein Structure Prediction Server, http://bioinf.cs.ucl.ac.uk/psipred). Predictions show that PrSUTs contain 12 transmembrane domains and both N- and C-termini on the cytoplasmic side. For all PrSUTs, amino acid length of N- and C-termini and the central cytoplasmic loop (between transmembrane segments VI and VII) are presented in a table.

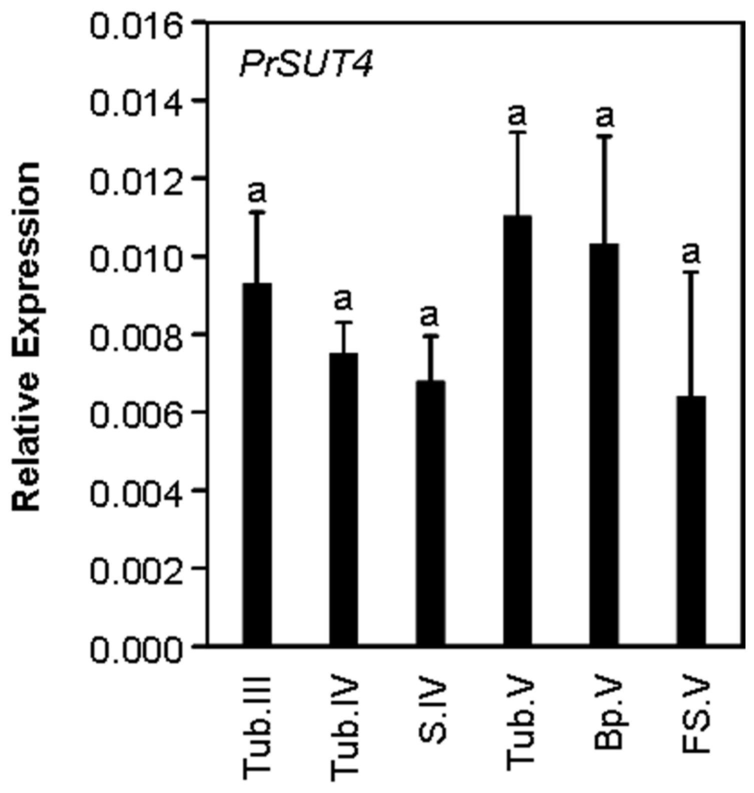

Figure S2. Development-related changes in the levels of *PrSUT4* transcripts in *Phelipanche ramosa*.

Sequence information from Phelipanche aegyptiaca contained in the Parasitic Plant Genome Project (PPGP; http://ppgp.huck.psu.edu) database reveals the existence of a fourth partial cDNA of *SUT* (OrAeBC1_18026: 402 pb), it is designed as *PrSUT4* in our study.

PrSUT4 transcripts accumulation is expressed relative to *EF1-α1* transcript levels. Based on OrAeBC1_18026 sequence, gene-specific primers were designed (forward, 5'-ATTCCACAGGTTATTGTGTCACTTG-3' and reverse, 5'-AGTTGCCGCCACCAAATAGT-3').

Data are means ± SE (n=3). Values with the same letter are not significantly different (ANOVA, SNK test, $P<0.05$).

Developmental stages are parasitic stages following attachment to host roots: growing tubercle (Tub.III); tubercle (Tub.IV) bearing the growing subterranean shoot (S.IV); tubercle (Tub.V) bearing the flowering shoot (following emergence); growing flowering shoot (apical part) (FS.V). Basal part (Bp.V) does not bear flowers and is larger and more fibrous than the flowering shoot (see Draie et al., 2011 for photographs).
